# Is there an association between ageist attitudes and frailty?

**DOI:** 10.1186/s12877-019-1357-y

**Published:** 2019-11-27

**Authors:** Douglas Salguero, Juliana Ferri-Guerra, Nadeem Y. Mohammed, Dhanya Baskaran, Raquel Aparicio-Ugarriza, Michael J. Mintzer, Jorge G. Ruiz

**Affiliations:** 1 Department of Public Health Sciences, University of Miami Miller School of Medicine, Miami, USA; 2 Miami VAHS GRECC Veterans Successful Aging for Frail Elders (VSAFE), Miami, USA; 3 Florida International University, Herbert Wertheim College of Medicine, Miami, USA; 4 Department of Medicine, University of Miami Miller School of Medicine, Miami, USA

**Keywords:** Frailty, Ageism, Implicit association test, Veterans

## Abstract

**Background:**

Frailty is defined as a state of vulnerability to stressors that is associated with higher morbidity, mortality and healthcare utilization in older adults. Ageism is “a process of systematic stereotyping and discrimination against people because they are old.” Explicit biases involve deliberate or conscious controls, while implicit bias involve unconscious processes. Multiple studies show that self-directed ageism is a risk factor for increased morbidity and mortality. The purpose of this study was to determine whether explicit ageist attitudes are associated with frailty in Veterans.

**Methods:**

This is a cross-sectional study of Veterans 50 years and older who completed the Kogan’s Attitudes towards Older People Scale (KAOP) scale to assess explicit ageist attitudes and the Implicit Association Test (IAT) to evaluate implicit ageist attitudes from July 2014 through April 2015. We constructed a frailty index (FI) of 44 variables (demographics, comorbidities, number of medications, laboratory tests, and activities of daily living) that was retrospectively applied to the time of completion of the KAOP and IAT. Odds ratios (ORs) and 95% confidence intervals (CIs) were calculated by multinomial logistic regression models with frailty status (robust, prefrail and frail) as the outcome variable, and with KAOP and IAT scores as the independent variables. Age, race, ethnicity, median household income and comorbidities were considered as covariates.

**Results:**

Patients were 89.76% male, 48.03% White, 87.93% non-Hispanic and the mean age was 60.51 (SD = 7.16) years. The proportion of robust, pre-frail and frail patients was 11.02% (*n* = 42), 59.58% (*n* = 227) and 29.40% (*n* = 112) respectively. The KAOP was completed by 381 and the IAT by 339 participants. In multinomial logistic regression, neither explicit ageist attitudes (KAOP scale score) nor implicit ageist attitudes (IAT) were associated with frailty in community dwelling Veterans after adjusting for covariates: OR = .98 (95% CI = .95–1.01), *p* = .221, and OR:=.97 (95% CI = .37–2.53), *p* = .950 respectively.

**Conclusions:**

This study shows that neither explicit nor implicit ageist attitudes were associated with frailty in community dwelling Veterans. Further longitudinal and larger studies with more diverse samples and measured with other ageism scales should evaluate the independent contribution of ageist attitudes to frailty in older adults.

## Background

Frailty is a state of vulnerability to stressors which is associated with higher morbidity, mortality and healthcare utilization in older adults [1]. The older Veteran population has a high prevalence of frailty [2]. Patients receiving care at Veteran healthcare institutions are older, sicker, functionally impaired, of lower socio-economic and educational status, and often uninsured and/or unemployed [3], risk factors known to be associated with frailty [4]. Prevention of frailty may depend on individuals’ positive attitudes aimed at preventing the future development of the syndrome in older age through chronic disease self-management and adoption of healthy lifestyle practices [5].

Ageism is defined as the systematic stereotyping of and discrimination against older adults [6]. Longitudinal studies demonstrate that having negative stereotypes and attitudes toward older adults at younger ages were associated years later with cardiovascular disease [7], memory impairment [8], decreased capacity to recover from disability [9], hearing loss [10], diminished will to live [11], lower participation in preventive activities [12], lower perception of functional health [13], poor recovery after myocardial infarction [14], increased risk for hospitalization [15], and increased mortality [16] compared with people who viewed old age more favorably. Many of these risk factors, conditions and outcomes have been associated with frailty [4]. Two theories may serve to explain these findings. The theory of stereotype embodiment proposes that lifetime exposures to ageism may lead to internalization of ageist messages by older persons which then become part of their unconscious beliefs [17]. The stereotype threat theory posits that under specific conditions involving these stereotypes, older persons would act subconsciously to fulfill those stereotypes, even if detrimental to themselves [18].

The purpose of this study was to determine in a sample of Veterans the association of explicit and implicit ageist attitudes with frailty. We predicted that ageist attitudes, explicit or implicit will be positively associated with frailty after adjustment for covariates that are known to be associated with this syndrome.

## Methods

### Design and participants

The present research is a retrospective, cross-sectional study of community-dwelling Veterans 50 years and older who were receiving outpatient care at a VA facility and previously completed a study of ageism. Between July 2014 and April 2015, these cognitively intact (Mini-Cog of > 3) and non-depressed (PHQ-2 of < 3) veterans were recruited into a study to measure implicit and explicit biases. Cognitive impairment and depression [19] are strongly associated with frailty and may be confounders for the effects of ageism [20]**.** This follow-up study used the data from this population, applied a frailty index and conducted a retrospective electronic health record review to determine frailty status of subjects and its association with implicit and explicit ageism biases. We obtained an expedited status from our IRB for this retrospective chart review which did not require obtaining an informed consent from participants.

Measures: Previously all study participants completed online versions of a socio-demographics questionnaire including questions about age, gender, race, and ethnic group and the following instruments:

#### Kogan’s attitudes toward old people scale (KAOP)

The scale is considered an explicit measure of attitudes toward older persons, which is easy to score and complete. The scale assesses participants’ general opinions and stereotypes about older adults and their intellectual abilities, image, levels of dependence, personality, living situation, personal appearance, influence and the individuals’ feelings of discomfort in the presence of older adults. The instrument consists of 17 matched pairs of positive and negative statements about older individuals. Individuals’ responses to the statements are rated on a 6-point Likert scale that ranges from “strongly agree” to “strongly disagree.” The possible scores range between 34 and 204 with higher scores representing more positive attitudes toward older individuals [21]. A score of 102 indicates a neutral attitude [22].

#### The Implicit Association Test (IAT)

Study participants completed an online version of implicit association test (IAT) created with the Inquisit software (Millisecond Software, Seattle, WA). The test asked participants to pair the terms “Old People” and “Young People” with “affective” attributes that were either positive (a total of 10 words) or negative (a total of 10 words) such as unpleasant-pleasant. As per IAT protocols, the words are combined with 10 photographs of older and 10 photographs of younger persons. In the next step, each participant completed an evaluative IAT [23], in which individuals paired pictures of old and young persons with pleasant and unpleasant words. First, participants completed two 10-trial practice blocks discriminating young from old faces, and pleasant from unpleasant words. The third and fourth blocks consisted of 20 trials each. Instructions for participants were to press one key (I/E) whenever they saw a pleasant word or a photograph of young person, and another key (I/E) whenever they saw an unpleasant word or a photograph of old person. The keys used to categorize young and old faces were exchanged in later blocks. The fifth block consisted of a 10-trial practice block in which participants were able to discriminate photographs of young from photographs of old faces using the new key assignments. The sixth and seventh blocks were comprised of 20 trials each. Participants were instructed to press one key (I/E) whenever they observed a photograph of an old person or a pleasant word, and another key (I/E) when they observed a photograph of a young person or an unpleasant word. Target category and attribute labels were always shown on the top left and top right of the screen respectively throughout the task, while words and stimulus photographs were shown at the center of the screen. A red “X” was shown whenever the participant made an error, which the participant was required to amend before moving onto the next trial. The software recorded latency periods in seconds to the correct response. Instructions for the participants were to classify the items as accurately and quickly as possible. The IAT is a timed word classification task which was scored according to protocols described by Greenwald et al. [24]. In this study, stronger associations of negative attributes with old people compared with young people revealed positive IAT d scores, whereas an IAT d score of 0 implied no differences in associations with young people compared with old people. IAT d scores were categorized into 5 categories: preference for older individuals (≤ .15), neutral (> − .15, ≤.15), slight (IAT d score > .15), moderate (IAT d score > .35), or strong (IAT d score ≥ .65) preference for younger individuals [24].

#### Frailty

In this follow-up study, for each patient, we matched the date of administration of study ageist attitudes assessments to a frailty index (FI) which was obtained from data available at the VA electronic health record and Corporate Data Warehouse (CDW). The FI was based on the deficit accumulation model of frailty and was calculated as a proportion of the number of factors (socio-demographic, medical and psychological conditions, laboratory tests, number of medications, blood pressure, body mass index and activities of daily living) present in over a total of 44 factors (see Supplementary Materials). A FI was calculated for each subject. At least 30 of 44 items were needed to calculate the FI and to be included in the study. The patients were stratified as robust (FI is ≤0.10), prefrail (FI between 0.10 and 0.20) or frail (FI is ≥0.20) [25].

### Data analysis

Baseline characteristics are presented as frequency (percent) for categorical variables, as mean ± SD for normally distributed continuous variables, and as median (interquartile range) for continuous variables with skewed distributions. Descriptive statistics included age, education, marital status, race, ethnicity, median household income, number of medications, body-mass index (BMI), and Charlson co-morbidity index. Median household income in the past 12 months (in 2011 inflation-adjusted dollars) by racial group from the U.S. Census Bureau, 2007–2011 was calculated using the 5-Digit ZIP Code Tabulation Area (ZCTA). We compared the mean scores using one-way ANOVA and comparisons of proportions were carried out using the Pearson chi-square test of homogeneity. Multinomial Logistic Regression Models using odds ratios (ORs) and 95% confidence intervals (CIs) were calculated with frailty status (robust, prefrail and frail) as the outcome variable, and with Kogan’s Attitudes Toward Old People Scale and IAT scores as independent variables. Age, race, ethnicity, median household income, and comorbidities were considered as covariates. A Pearson correlation was run to assess the relationship between Kogan’s Attitudes Toward Old People Scale and IAT. Associations were considered significant if *p* < 0.05. All analyses were performed using the SPSS 24.0 for Macintosh (SPSS, Inc., Chicago, Illinois) and SAS version 3.71 (SAS Institute Inc., Cary, North Carolina). All statistical tests were two-tailed, and statistical significance was assumed for a *p*-value < 0.05.

## Results

Patient Characteristics (see Table 1): Three hundred and eighty one participants had 30 factors or more needed for calculation of the FI: 48.03% White, 88.76% non-Hispanic and the mean age was 60.51 (SD = 7.16) years. The proportion of robust, pre-frail and frail patients was 11.02% (*n* = 42), 58.58% (*n* = 227) and 29.40% (*n* = 112) respectively. As seen in Table 1, frail older Veterans were less likely to be married, have higher levels of multimorbidity and were taking more medications than non-frail Veterans. All 381 participants completed the KAOP, 364 (95.50%) show a general positive attitude toward older people (scores > 102). Of the 381 total, 339 participants completed the IAT, scores showed that 22 (6.5%) preferred older people, 32 (9.40%) were neutral, 43 (12.70)%) had a slight, 63 (18.60%) moderate and 179 (52.80%) strong preference for younger individuals. There was no correlation between the KAOP and the IAT scores (r = .043, *p* = .431).

**Table 1:** Participant Characteristics Stratified by Frailty status

	Total (*n* = 381, 100%)	Robust (*n* = 42, 11.02%)	Prefrail (*n* = 227, 59.58%)	Frail (*n* = 112, 29.40%)	*P*
Age, mean (SD)	60.51 (7.16)	59.14 (7.31)	60.59 (7.40)	60.84 (6.60)	.408
Gender, n (%)	342 (89.76)	38 (90.48)	202 (88.99)	102 (91.07)	.827
Caucasian, n (%)	183 (48.03)	17 (40.48)	111 (48.90)	55 (49.11)	.583
Not Hispanic*, n (%)	335 (87.93)	34 (80.95)	199 (87.67)	102 (91.07)	.225
Married, n (%)	91 (23.90)	24 (57.10)^b^	51 (22.50)^a^	16 (14.30)^b^	**<.0001**
Median Household Income, mean $ (SD)	45,942 (18,375)	48,549 (19,226)	45,799 (18,562)	45,256 (17,742)	.603
Charlson Comorbidity Index, mean (SD)	3.80 (1.87)	2.74 (1.32)^a^	3.61 (1.70)^b^	4.56 (2.11)^c^	**<.0005**
More than 5 Medications, n (%)	195 (51.18)	3 (7.14)^a^	104 (45.81)^b^	88 (78.57)^c^	**<.0005**
Frailty Index, mean (SD)	.18 (.071)	.07 (.02)^a^	.16 (.03)^b^	.27 (.05)^c^	**<.0005**
Kogan Scores, mean (SD)	121.13 (11.90)	123.36 (12.52)	120.89 (13.13)	120.76 (8.59)	.457
IAT Scores*, mean (SD)	.6208 (.4746)	.6754 (.4575)	.6302 (.4606)	.58349 (.5091)	.744

*SD* standard deviation, *n* number of participants

*Available for 339 participants

Data with different superscript letters are significantly different *p* < 0.05, according to the post hoc ANOVA statistical analysis for continuous variables and chi square for categorical variables. The column means test table assigns a superscript letter (a, b or c) to the robust, prefrail and frail groups. If a pair of values is significantly different, the values have different subscript letters assigned to them. If a pair of values are not significantly different, the values will have the same superscript letters assigned to them. Data without superscripts is not significantly different between robust, prefrail and frail groups

Explicit Ageism: There were no significant differences in the Kogan’s Attitudes Toward Old People Scale scores between the groups (Table 1). In multinomial logistic regression, highest KAOP scores were not associated with frailty in unadjusted (OR = .98, 95%CI = .96–1.01, *p* = .232) or adjusted (OR = .98, 95%CI = .95–1.01, *p* = .221) models (Table 2).

**Table 2:** Estimated Odds Ratios and 95% Confidence Intervals for “Prefrail” and “Frail” Categories Relative to Robust by Kogan and Implicit Association Test scores

Measure (Scores)	“Prefrail” relative to “Robust”*	*p* value	“Frail” relative to “Robust”*	*p* value
Kogan Scores				
Unadjusted	.98 (.96–1.01)	.219	.98 (.96–1.01)	.232
Adjusted	.98 (.96–1.01)	.253	.98 (.95–1.01)	.221
Implicit Association Test Scores*				
Unadjusted	.81 (.36–1.81)	.608	.66 (.28–1.55)	.339
Adjusted	1.25 (.51–3.06)	.619	.97 (.37–2.53)	.950

*Data available for 339 participants

*Models adjusted for age, race, ethnicity, education, median household income and comorbidities. Data for the adjustment was missing in XX patients (Y for ethnicity)

Implicit Ageism: There were no significant differences in the Implicit Association Test scores between the groups (Table 1). Likewise, there were no differences in the proportion of individuals with strong preferences for younger people (IAT ≥ .65) between robust, prefrail and frail groups: 54.50% (*n* = 18), 53.70% (*n* = 110) and 50.50% (*n* = 51), *p* = .854 respectively, IAT scores were not associated with frailty in unadjusted (OR = .66, 95%CI = .28–1.55, *p* = .339) or fully adjusted (OR = .97, 95%CI = .37–2.53, *p* = .950) models (Table 2).

## Discussion

Our hypothesis that older Veterans’ ageist attitudes after adjustment for confounders would be associated with the frailty syndrome was rejected. Explicit ageism as measured with the Kogan’s Attitudes Toward Old People Scale (KAOP) and implicit ageism as measured with the Implicit Association Test (IAT) were not associated with a greater risk for frailty as we predicted. Neither explicit nor implicit ageism were associated with an increased risk for frailty. The KAOP and IAT scores were not correlated. Although most participants showed a favorable explicit attitude toward older adults, most participants showed negative implicit bias toward older individuals. To the best of our knowledge, this is the first study that explores the cross-sectional association of ageist attitudes with frailty.

The literature regarding individuals’ positive attitudes towards aging is scant. Most studies address health care professionals’ attitudes towards older adults [26] but few address the attitudes of older patients themselves toward their own aging process. The overall positive attitudes towards aging shown by our older participants is consistent with previous studies [27, 28] and may partially explain the lack of association of ageism with frailty in our study. A large cross-sectional study in 20 countries revealed that older persons who were satisfied with their own health displayed more positive attitudes towards the physical and psychosocial aspects of their own aging [28]. Older individuals with positive aging attitudes were more likely to adhere to healthy behaviors [29]. Positive attitudes to aging may be associated with higher life satisfaction and self-reported physical and mental health [27, 28], factors that may potentially contribute to reduce the risk of frailty. Future studies should examine the complex relationship between satisfaction with own health, ageist attitudes, health behaviors and frailty.

The lack of association between ageist attitudes and frailty may be explained by some of the characteristics of the study sample. The mean age of this sample is sixty which may be early in the course of frailty for most participants. Frailty is more common with older age and in women; conceivably, in a sample with more older women, an association may occur. The KAOP scale assesses individuals’ attitudes toward typical older adults on several domains, it does not evaluate the domain of personal attitudes to oneself as other scales. This dissociation between attitudes toward one’s own aging as compared to other older persons has been reported [30, 31]. Measures that focus on self-perceptions of aging domains may be more likely associated with frailty and conditions associated with frailty as shown by others [7–16]. In terms of implicit attitudes, Levy et al. has shown in experimental studies that individuals’ exposure to subliminal negative implicit primes was associated with behavioral, psychological and physiological deleterious changes [11, 32, 33] which if persistent and long-lasting may at least theoretically be associated with the eventual development of frailty. Another factor is that despite their high multimorbidity burden veterans have access to an integrated healthcare system that provides a range of medical and social services which may ameliorate the possible negative effects of ageist attitudes on their health. Efforts in an integrated healthcare system may ensure adequate support for patient self-management activities, enhanced communication, and care coordination programs within a mature patient centered medical home model [34].

Our sample is relatively younger than that of most other studies using the frailty index. The Canadian Health Measures Study, using the frailty index estimated a prevalence of frailty of 20.2% in adults 50–65 years old [35], which is lower that the 29.40% we found. However, the prevalence of frailty is our study is comparable to that found in the largest, nationwide prevalence study of US Veterans receiving care at Department of Veterans Affairs (VA) Medical Centers [2]. US Veterans have lower socioeconomic and educational status, increased rates of disability, multimorbidity, mental illness, social isolation, substance abuse, and homelessness, variables often associated with frailty [3, 36].

Strengths of this study include the large number of Veterans with thorough assessments of explicit and implicit ageist attitudes as well as the use of a validated process to calculate the FI incorporating comprehensive electronic health record data. There are a few limitations. Our participants were part of a convenience instead of a randomly selected sample. The study was also limited to patients at one VA medical center who may be different in from other Veterans’ facilities in the US in terms of ethnic, racial, educational, and socio-economic characteristics. The sample was predominantly White and male which could limit the generalizability of the findings to other racial and ethnic groups as well as females. The cross-sectional design of our study may limit our conclusions about the causal effect of explicit and implicit ageism on frailty. Nonetheless, our results and conclusions may have important clinical implications for the field of ageism research encouraging future research. Future research may benefit from investigating whether explicit and implicit ageism predicts frailty in longitudinal studies including assessment of attitudes toward their own aging process, including more diverse samples, and studying the influence on frailty of the interaction between individuals holding ageist attitudes and their healthcare systems.

## Conclusions

The study reveals that neither explicit nor implicit ageist attitudes were associated with frailty after adjustment for covariates. Future research may wish to examine differences between older individuals from more diverse samples and as part of longitudinal studies.

## Data Availability

The datasets used and/or analyzed during the current study are available from the corresponding author on reasonable request.

## Abbreviations

FI: Frailty Index
IAT: The Implicit Association Test
KAOP: Kogan’s Attitudes Toward Old People Scale
